# Comparison of cytokine/chemokine profiles between dermatomyositis and anti-synthetase syndrome

**DOI:** 10.3389/fneur.2022.1042580

**Published:** 2022-12-08

**Authors:** Yikang Wang, Yiming Zheng, Yawen Zhao, Yilin Liu, Wenhao Zhang, Meng Yu, Zhiying Xie, Hongjun Hao, Feng Gao, Wei Zhang, Zhaoxia Wang, Yun Yuan

**Affiliations:** ^1^Department of Neurology, Peking University First Hospital, Beijing, China; ^2^Beijing Key Laboratory of Neurovascular Disease Discovery, Beijing, China

**Keywords:** dermatomyositis, anti-synthetase syndrome, cytokines, chemokines, differential diagnosis

## Abstract

**Objectives:**

Dermatomyositis (DM) and anti-synthetase syndrome (ASS) are autoimmune diseases with multisystem involvement. Despite sharing some clinical and myopathological features, these are two diseases with different pathogeneses and prognoses. We aimed to clarify and compare cytokine/chemokine profiles in both disorders, which may help in the differential diagnosis.

**Materials and methods:**

We collected clinical data and serum samples of consecutive patients with DM and ASS. Quantibody^®^ Human Inflammation Array 3 for cytokines/chemokines was performed in the serum of all participants. Receiver operating characteristic analysis with the area under the curve and Youden's index were performed.

**Results:**

Eight newly diagnosed and treatment-naïve patients with DM, nine newly diagnosed and treatment-naïve patients with ASS, and 14 healthy controls were enrolled. Serum C-C motif chemokine ligand (CCL) 2, CCL4, C-X-C motif chemokine ligand (CXCL) 13, and tumor necrosis factor receptor 2 (TNFR2) were increased in patients with both DM and ASS. Serum interleukin (IL)-1 receptor type 1 (IL-1ra), IL-1b, CCL1, CXCL11, and CCL3 were modulated in patients with DM only, and IL-8, CXCL9, and tissue inhibitors of metalloproteinases-1 (TIMP-1) in patients with ASS only. Serum CCL2, CXCL13, and TNFR2 accurately distinguished patients with DM and ASS from healthy controls, as shown by the area under the curve >0.80. Moreover, receiver operating characteristic analysis showed that, as biomarkers for discrimination between DM and ASS, the combination of IL-1ra and TIMP-1, had an area under the curve of 0.944, a sensitivity of 87.5%, and a specificity of 88.9%.

**Conclusion:**

Our study demonstrated that serum levels of cytokines/chemokines showed a different pattern in newly diagnosed patients with DM and ASS, in which serum IL-1ra and TIMP-1 could be used to distinguish between the two diseases.

## Introduction

Dermatomyositis (DM) is a type I interferonopathy characterized by inflammation of small vessels mainly involving the skin and skeletal muscles. Characteristic skin manifestations include Gottron's papules/sign, periungual erythema, heliotrope rash, V sign, and shawls sign, whereas the spectrum of muscle involvement ranges from no or little to severe weakness (1–4). Additionally, some patients may present with other systemic manifestations, such as interstitial lung disease (ILD) or malignancy (2). There are five myositis-specific antibodies (MSAs) in DM, including anti-transcription intermediary factor 1-γ, anti-complex nucleosome remodeling histone deacetylase, anti-melanoma differentiation gene 5, anti-nuclear matrix protein 2 (NXP2), and anti-small ubiquitin-like modifier-activating enzyme (1, 2). The common myopathological changes in DM are perifascicular atrophy (PFA), regional muscle edema, perimysial infiltrates, capillary loss, and sarcoplasmic myxovirus resistance protein A (MxA) expression (1, 2, 5, 6).

Anti-synthetase syndrome (ASS), characterized by myositis, ILD, skin rash, arthropathy, Raynaud phenomenon, and mechanic's hands, is a rare chronic autoimmune disease (7, 8). Eight antibodies targeting cytoplasmic aminoacyl-tRNA synthetases have been recognized, including anti-Jo-1 (histidyl), anti-PL-7 (threonyl), anti-PL-12 (alanyl), anti-EJ (glycyl), anti-OJ (isoleucyl), anti-KS (asparaginyl), anti-Zo (phenylalanyl), and anti-Ha (tyrosyl) (9). The myopathological features in ASS are PFA, perifascicular necrosis (PFN) and regeneration, perimysial fragmentation, major histocompatibility complex class II (MHC-II) staining of the perifasciculum, and absence of MxA upregulation (6, 7).

Dermatomyositis and anti-synthetase syndrome are both multisystemic autoimmune diseases, and they share some clinical and pathological manifestations. It is often difficult to distinguish these two diseases clinically and pathologically in the absence of MSAs. Nonetheless, correct diagnosis is fundamental, because they have different prognoses and responses to therapies (10–12). Although sarcoplasmic MxA expression has a high sensitivity for the diagnosis of DM (71%−77%) (5, 13), muscle biopsy is not yet fully available. Despite the increasing number of studies on serum levels of cytokines/chemokines in patients with DM and ASS (14–16), there is still no consensus on the differences in these mediators between these two diseases. The identification of biomarkers could lead to a better understanding of the pathogenesis of diseases and discrimination between DM and ASS. Therefore, we aimed to clarify and compare cytokine/chemokine profiles in newly diagnosed patients with DM and ASS.

## Materials and methods

### Patients

We collected data from eight newly diagnosed and treatment-naïve patients with DM and nine newly diagnosed and treatment-naïve patients with ASS. DM was diagnosed according to the 239th European Neuro Muscular Center International Workshop guidelines (1). ASS was diagnosed with definitive serology findings of one of the anti-aminoacyl-tRNA synthetases antibodies tested, along with at least one triad finding, including myositis, arthritis, and ILD (3). Demographic and clinical data were retrospectively collected through patients' first medical records. Muscle weakness was assessed by manual muscle strength (Medical Research Council scale, MRC), with severe weakness defined as the grade of the weakest muscle ≤3/5. Serum levels of creatine kinase (CK) were analyzed as routine tests. The presence of ILD was determined by high-resolution computed tomography findings. Disease activity was evaluated using Myositis Disease Activity Assessment Tool (MDAAT).

In the present study, 14 healthy controls (HCs) were included. The exclusion criteria for HCs were neuromuscular disease, autoimmune disease, and infectious disease. The male-to-female ratio was 8:6, and the mean age was 29 (interquartile range 24–41.8) years.

### Serum antibodies detection

Myositis-specific antibodies and myositis-associated antibodies (IgG antibodies against 24 different cytoplasmic/nuclear antigens) were detected using commercial line immunoblot assay (EUROIMMUNE, Lubeck, Germany). The results were read by two independent observers and were considered positive if the bands showed moderate or strong reactivity (8).

### Muscle pathology

Muscle biopsy samples were taken from the biceps brachii or quadriceps femoris of eight patients with DM and nine patients with ASS before the initiation of immunosuppressive treatment. Open muscle biopsies were performed on all patients after written informed consent. Serial frozen sections were stained with hematoxylin and eosin (HE), modified Gomori trichrome, periodic acid-Schiff, oil red O, adenosine triphosphate enzyme (pH 4.5 and 10.8), nicotinamide adenine dinucleotide tetrazolium reductase (NADH-TR), succinate dehydrogenase, and cytochrome c oxidase stains. The sections were stained for immunohistochemistry with primary antibodies against human dystrophin, sarcoglycan, dysferlin, CD3, CD4, CD8, CD20, CD68, major histocompatibility complex class I (MHC-I), membrane attack complex (MAC), and MxA.

A modified histopathological muscle biopsy scoring system was established according to the method developed by Tanboon et al. (17) (Supplementary Table 1). First, specimens were divided into four domains: muscle fiber, inflammation, connective tissue, and vascular domain. For each domain, the score was calculated based on its components. The muscle fiber domain included the total score of necrotic fiber, regenerating fiber, atrophic fiber away from the perifascicular area, perifascicular atrophy, and fiber with internalized nuclei. The inflammation domain contained the total score of infiltrated CD3^+^, CD20^+^, and CD68^+^ cells in muscle tissue scored from 0 to 2 for each item. The connective tissue domain was scored as 1 or 0 according to the presence or absence of corresponding lesions. The vascular domain included the total score of arterial abnormality, infarction, and MAC^+^ capillaries. The total score of the muscle biopsy was obtained by summing each domain score. The scoring process was performed by two individual physicians (WZ and YY) and their mean was used for the final scores.

### Measurement of serum cytokines and chemokines

The cytokine/chemokine expression was screened in all participants, using Quantibody^®^ Human Inflammation Array 3 (RayBiotech Inc., Norcross, GA), according to the manufacturer's instructions. All patients were treatment-naïve. A panel of 40 cytokines/chemokines was analyzed: C-X-C motif chemokine ligand (CXCL) 8, CXCL9, CXCL11, CXCL13, C-C motif chemokine ligand (CCL) 1, CCL2, CCL3, CCL4, CCL5, CCL15, CCL24, granulocyte colony-stimulating factor, macrophage colony-stimulating factor, granulocyte-macrophage colony-stimulating factor, interleukin (IL)-1α, IL-1b, IL-1 receptor type 1 (IL-1ra), IL-2, IL-4, IL-5, IL-6, IL-6 receptor subunit alpha, IL-7, IL-10, IL-11, IL 12 p40 mutant, IL 12 p70 mutant, IL-13, IL-15, IL-16, IL-17A, tumor necrosis factor (TNF)-α, TNF-β, TNF receptor superfamily member 1A, TNF receptor 2 (TNFR2), tissue inhibitor of metalloproteinases-1 (TIMP-1), TIMP-2, intercellular adhesion molecule 1, interferon gamma, and platelet-derived growth factor BB.

### Bioinformatics analysis

Raybiotech software was used to remove the background, normalize the data, and draw standard curves. Processed data were used as standard data for analysis. The R software package limma from R/Bioconductor was used to compare serum levels of cytokines/chemokines between two groups using moderated *t*-statistics. The results included log_2_ (Foldchange) (log_2_FC), logFC, and *P*-value for each factor. Next, the false discovery rate (the Benjamini–Hochberg method) was used to calculate the adjusted *P*-value. Differentially expressed cytokines/chemokines were defined as those with adjusted *P*-value < 0.05 and FC >1.2 or <0.83 (absolute logFC > 0.263) (18–20). Heat maps were drawn to screen for and depict the logical connections between differentially expressed cytokines/chemokines. Heat map analysis was executed using R/Bioconductor software (R version 4.0.2; Bioconductor version 3.12) and Metascape (https://metascape.org).

### Statistical analysis

Qualitative data, including sex, presence of symptoms, positive antinuclear antibody, and presence of histopathological features are presented as frequencies and percentages. Quantitative data are presented as median (interquartile range; IQR). Comparisons of clinical and histopathological indicators between the two groups were performed using the Mann–Whitney *U*-test for quantitative data and Fisher exact test for qualitative data. Spearman's correlation tests were used to assess the relationship between variables. Receiver operating characteristic (ROC) analysis with the area under the curve (AUC) was performed for non-paired samples. Optimal cutoff points were identified by assessing the maximum sum of sensitivity and specificity, according to the Youden Index. *P* < 0.05 was considered statistically significant.

## Results

### Clinical features

The main demographic and clinical characteristics of eight patients with DM (two men and six women) are shown in Table 1. The median age at disease onset was 24.9 (IQR 4.3–56) years. The median disease duration from disease onset to the time of diagnosis was 5 (IQR 1.8–33) months. Six patients were diagnosed with classic DM, whereas the remaining two were diagnosed with amyopathic DM. All patients had DM-specific cutaneous manifestations with heliotrope rash, Gottron's papules/sign, V sign, and/or shawls sign. Limb weakness was observed in 6/8 (75.0%) patients, and three of them presented with severe weakness. The median MRC total score was 40 (IQR 36.8–43.8). Neck weakness and dysphagia appeared in 5/8 (62.5%) patients and 1/8 (12.5%) patient, respectively; and 4/8 (50.0%) patients presented with myalgia. Fever occurred in 4/8 (50.0%) patients. Arthralgia, Raynaud phenomenon, ILD, and other autoimmune diseases were not observed in any of the patients. The median serum CK was 395.5 (IQR 118.3–3977.8) IU/L. Serum anti-NXP2 antibody was positive in all patients with DM.

**Table 1:** Demographic and clinical features of patients with DM and ASS.

**Features**	**DM (*n* = 8)**	**ASS (*n* = 9)**	***P-*value**
Female, *n* (%)	6 (75.0%)	5 (55.6%)	0.620
Age at disease onset, median (IQR), years	24.9 (4.3, 56)	40 (24.2, 53)	0.606
Disease duration, median (IQR), months	5 (1.8, 33)	5 (1, 23)	0.963
Muscle weakness, *n* (%)	6 (75.0%)	7 (77.8%)	>0.999
Limb weakness, *n* (%)	6 (75.0%)	7 (77.8%)	>0.999
Neck weakness, *n* (%)	5 (62.5%)	4 (44.4%)	0.637
Severe weakness, *n* (%)	3 (37.5%)	3 (33.3%)	>0.999
Dysphagia, *n* (%)	1 (12.5%)	2 (22.2%)	>0.999
Myalgia, *n* (%)	4 (50.0%)	5 (55.6%)	>0.999
Extramuscular symptoms			
Fever, *n* (%)	4 (50.0%)	1 (11.1%)	0.131
Skin rash, *n* (%)	8 (100.0%)	4 (44.4%)	0.029^*^
Arthralgia, *n* (%)	0 (0.0%)	5 (55.6%)	0.029^*^
Raynaud phenomenon, *n* (%)	0 (0.0%)	1 (11.1%)	>0.999
Interstitial lung disease, *n* (%)	0/5 (0.0%)	4/7 (57.1%)	0.081
Blood examination			
Serum CK, median (IQR), IU/L	395.5 (118.3, 3977.8)	1066.9 (63.0, 6091.0)	0.888
Positive ANA, *n* (%)	2/3 (66.7%)	5/7 (71.4%)	>0.999
MRC total scores, median (IQR)	40 (36.8, 43.8)	41 (34.5, 43.5)	0.888
MDAAT scores, median (IQR)	9.5 (8, 15.5)	9 (5, 10)	0.236

DM, dermatomyositis; ASS, anti-synthetase syndrome; CK, creatine kinase; ANA, antinuclear antibody; MRC, Medical Research Council; MDAAT, Myositis Disease Activity Assessment Tool; IQR, interquartile range.

* P < 0.05.

The main demographic and clinical characteristics of nine patients with ASS (four men and five women) are also shown in Table 1. The median age at disease onset was 40 (IQR 24.2–53) years and the median disease duration from disease onset to the time of diagnosis was 5 (IQR 1–23) months. A total of four out of nine (44.4%) patients had typical DM skin lesions with heliotrope rash and Gottron's papules/sign, and two of them presented with Mechanics' hands. Limb weakness was observed in 7/9 (77.8%) patients, and three of them had severe weakness. The median MRC total score was 41 (IQR 34.5–43.5). Neck weakness was observed in 4/9 (44.4%) patients, and two of these patients also showed dysphagia. A total of five out of nine (55.6%) patients had myalgia. Other symptoms were arthralgia (five cases), ILD (four cases), and the Raynaud phenomenon (one case). The median serum CK was 1,066.9 (IQR 63.0–6091.0) IU/L. Serum anti-Jo-1 antibody was positive in 5/9 (55.6%) patients and anti-EJ antibody in 4/9 (44.4%) patients. The median MDAAT score was 9 (IQR 5–10).

Most clinical features showed no significant difference, except for a higher percentage of skin rash in DM than ASS (100 vs. 44.4%, *P* = 0.029), and a lower percentage of arthralgia in DM than ASS (0 vs. 55.6%, *P* = 0.029).

### Histopathological features

Among all eight muscle specimens from patients with DM, 2/8 (25.0%) patients showed perimysial fragmentation (Table 2), while 4/8 (50.0%) patients had focal perimysial inflammatory infiltrates. PFA was observed in 4/8 (50.0%) patients and none showed PFN. Punched-out fibers appeared in 1/8 (12.5%) patient on NADH-TR staining. A total of 6 out of 8 (75.0%) patients had COX-negative/decreasing fibers in perifascicular region. MHC-I showed diffuse upregulation in 3/8 (37.5%) patients, diffuse upregulation with perifascicular enhancement in 2/8 (25.0%) patients, and focal upregulation in perifascicular regions in 1/8 (12.5%) patients. MAC deposition on the endomysial capillaries was observed in 5/8 (62.5%) patients; two of these patients also showed deposition on non-necrotic myofibers. MxA-positive myofibers appeared in 6/8 (75.0%) patients, all of whom were patients with classic DM. The median pathological total score was 16.5 (IQR 4.5–22.5).

**Table 2:** Histopathological findings of patients with DM and ASS.

	**DM (*n* = 8)**	**ASS (*n* = 9)**	***P*-value**
Histopathological features
Perimysial fragmentation, *n* (%)	2 (25.0%)	3 (33.3%)	>0.999
Focal perimysial inflammatory infiltrates, *n* (%)	4 (50.0%)	4 (44.4%)	>0.999
Perifascicular fiber atrophy, *n* (%)	4 (50.0%)	3 (33.3%)	0.637
Perifascicular necrosis, *n* (%)	0 (0.0%)	7 (77.8%)	0.002^**^
Punched out fibers, *n* (%)	1 (12.5%)	0 (0.0%)	0.471
COX-negative/decreasing fibers in perifascicular region, *n* (%)	6 (75.0%)	3 (33.3%)	0.153
MHC-I expression, *n* (%)	6 (75.0%)	6 (66.7%)	>0.999
Diffuse in muscle fiber membrane, *n* (%)	3 (37.5%)	2 (22.2%)	0.620
Diffuse in muscle fiber membrane with perifascicular enhancement, *n* (%)	2 (25.0%)	4 (44.4%)	0.620
Expression in perifascicular regions	1 (12.5%)	0 (0.0%)	>0.999
MAC expression, *n* (%)	5 (62.5%)	9 (100.0%)	0.082
Necrotic fibers, *n* (%)	0 (0.0%)	3 (33.3%)	0.206
Non-necrotic myofiber, *n* (%)	2 (25.0%)	6 (66.7%)	0.153
Capillaries, *n* (%)	5 (62.5%)	6 (66.7%)	>0.999
MxA-positive myofibers, *n* (%)	6 (75.0%)	0/5 (0.0%)	0.021^*^
Pathological scores			
Muscle fiber domain, median (IQR)	2.5 (1.0, 4.0)	3.5 (1.3, 6.5)	0.279
Inflammation domain, median (IQR)	0.5 (0.0, 2.8)	0.5 (0.0, 1.0)	0.798
Connective tissue domain, median (IQR)	1.5 (1.0, 2.0)	1.0 (0.3, 1.8)	0.645
Vascular domain, median (IQR)	10.5 (2.3, 15.3)	5.0 (3.5, 12.5)	0.442
Total scores, median (IQR)	16.5 (4.5, 22.5)	9.0 (6.3, 20.8)	>0.999

DM, dermatomyositis; ASS, anti-synthetase syndrome; COX, cytochrome c oxidase; MHC-I, major histocompatibility complex class I; MAC, membrane attack complex; MxA, myxovirus resistance protein A. IQR, interquartile range.

* P < 0.05.

** P < 0.01.

Among all nine muscle specimens from patients with ASS, 3/9 (33.3%) patients showed perimysial fragmentation and 4/9 (44.4%) patients had focal perimysial inflammatory infiltrates. PFN appeared in 7/9 (77.8%) patients, three of them showing PFA. COX-negative/decreasing fibers were present in 3/9 (33.3%) patients. MHC-I showed diffuse upregulation in 2/9 (22.2%) patients and diffuse upregulation with perifascicular enhancement in 4/9 (44.4%) patients. MAC deposition on non-necrotic myofibers was found in 6/9 (66.7%) patients, and three of these patients also showed deposition on the necrotic myofibers. MAC deposition on the endomysial capillaries occurred in 6/9 (66.7%) patients. Sarcoplasmic MxA expression was not detected in any patient with ASS. The median pathological total score was 9.0 (IQR 6.3–20.8).

**Figure 1:**
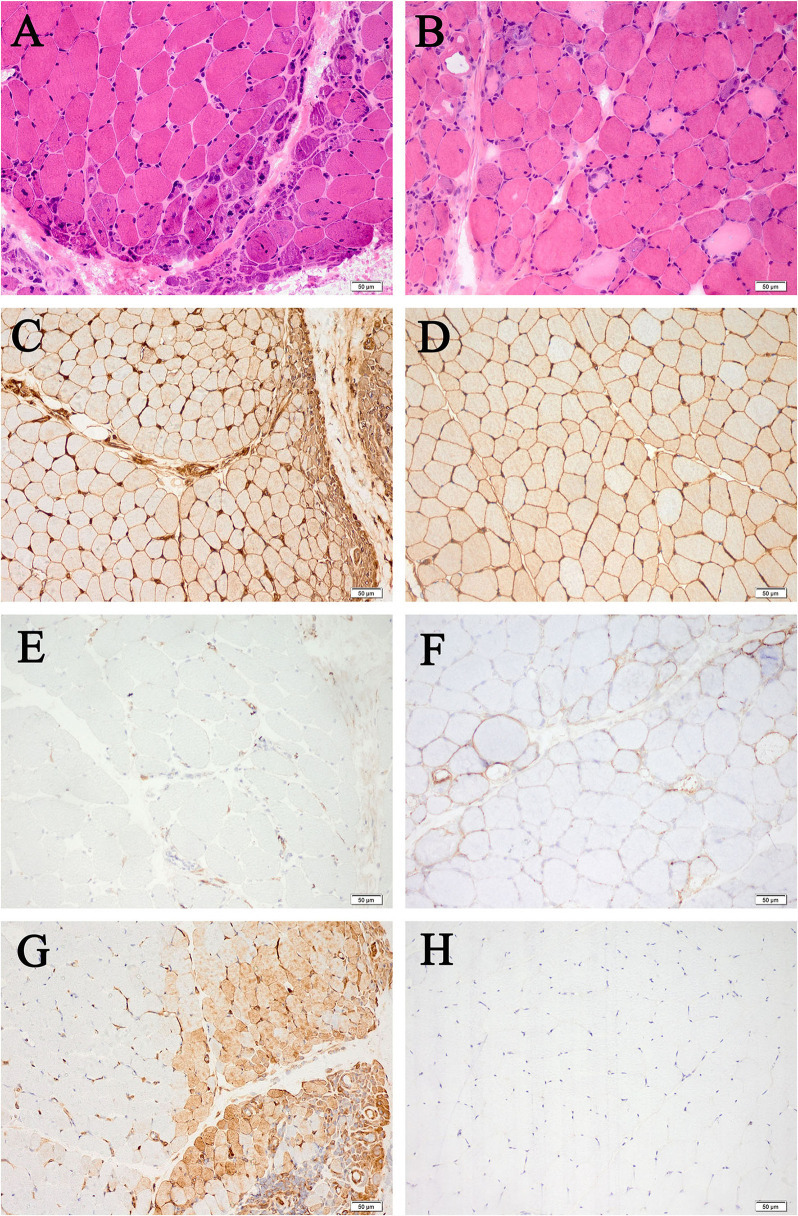
Myopathological changes in patients with DM **(A,C,E,G)** and patients with ASS **(B,D,F,H)** (200× magnification). **(A)** Perifascicular fiber atrophy on HE staining. **(B)** Perifascicular necrosis on HE staining. **(C)** Diffuse MHC-I upregulation, with enhancement in the perifascicular region. **(D)** Diffuse MHC-I upregulation. **(E)** MAC deposition on the endomysial capillaries. **(F)** Sarcolemma MAC deposition on non-necrotic fibers. **(G)** MxA-positive fibers. **(H)** MxA-negative fibers. DM, dermatomyositis; ASS, anti-synthetase syndrome; HE, hematoxylin and eosin; MHC-I, major histocompatibility complex class I; MAC, membrane attack complex; MxA, myxovirus resistance protein A.

Most histopathological features showed no significant difference, except for a higher percentage of MxA-positive fibers in DM than ASS (75 vs. 0%, *P* = 0.021), and a lower percentage of PFN in DM than ASS (0 vs. 77.8%, *P* = 0.002). Myopathological changes in patients with DM and ASS are shown in Figure 1.

### Cytokine/chemokine profiles

**Figure 2:**
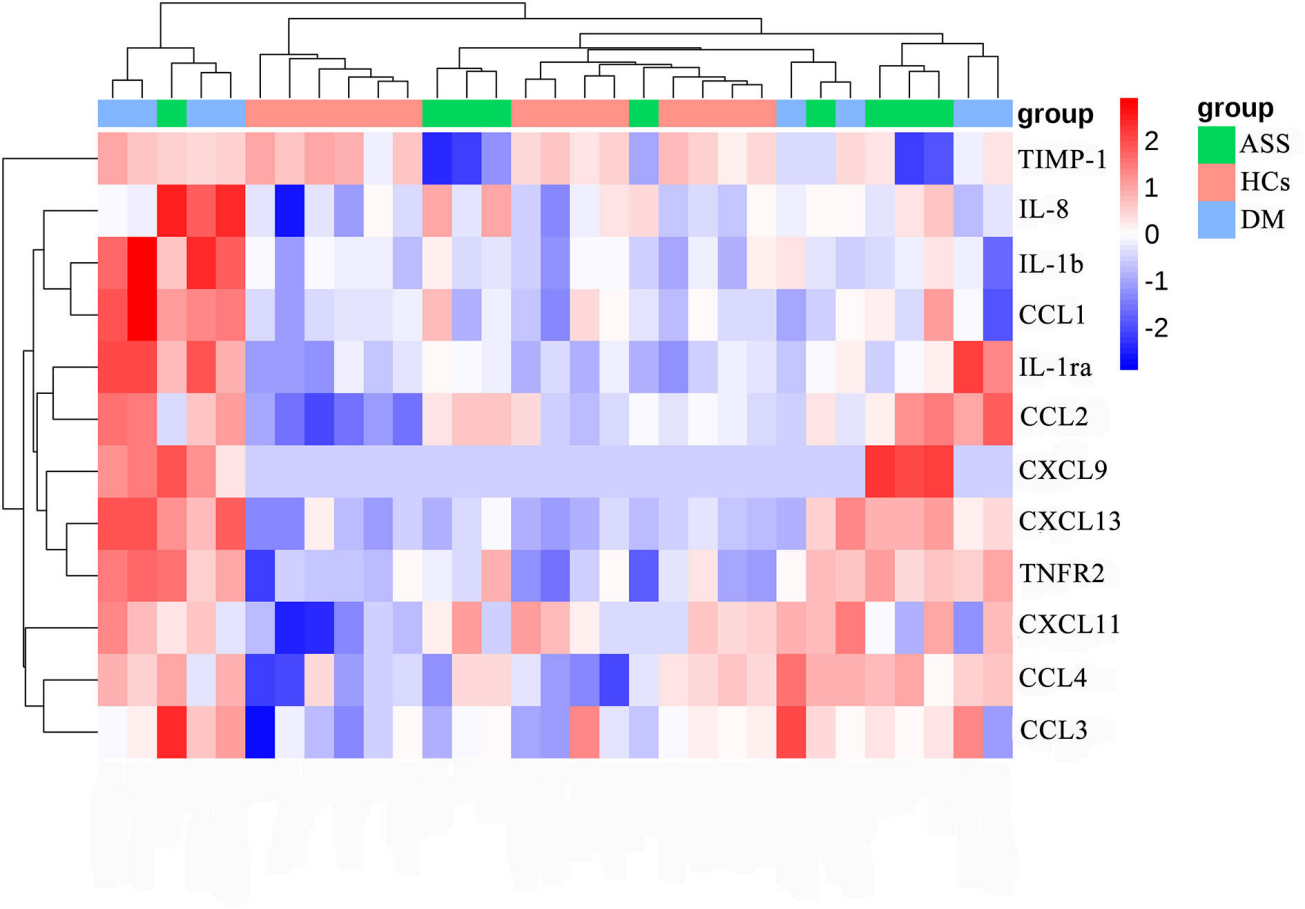
Heat map of differentially expressed cytokines/chemokines. DM, dermatomyositis; ASS, anti-synthetase syndrome; HCs, healthy controls.

At baseline, serum levels of CCL2, CCL4, TNFR2, CXCL13, IL-1ra, IL-1b, CCL1, CXCL11, and CCL3 were significantly higher in patients with DM than HCs (adjusted *P*-value < 0.05; FC > 1.2 or < 0.83; Table 3, Figure 2, and Supplementary Figures 1A–I). At baseline, higher serum levels of CCL2, CCL4, TNFR2, CXCL13, IL-8, and CXCL9 were detected in patients with ASS than in the HCs, except for a significant reduction in serum level of TIMP-1 (adjusted *P*-value < 0.05; FC > 1.2 or < 0.83; Table 3, Figure 2, Supplementary Figures 1A–D,J–L).

**Table 3:** Foldchange and diagnostic performance of serum cytokines/chemokines in DM and ASS.

**Cytokines/chemokines**	**DM vs. HCs**		**ASS vs. HCs**		**Area under the curve**	
	**Adjusted *P*-value**	**Foldchange**	**Adjusted *P*-value**	**Foldchange**	**DM vs. HCs**	**ASS vs. HCs**
CCL2	< 0.001^*^	1.910^*^	0.017^*^	1.656^*^	0.911	0.929
CCL4	< 0.001^*^	2.349^*^	0.022^*^	1.849^*^	0.929	0.778
CXCL13	< 0.001^*^	3.568^*^	0.043^*^	2.163^*^	0.929	0.841
TNFR2	< 0.001^*^	1.571^*^	0.022^*^	1.347^*^	0.982	0.841
IL-1ra	< 0.001^*^	3.656^*^	0.147	1.464	0.937	0.865
IL-1b	0.002^*^	2.522^*^	0.431	1.266	0.732	0.611
CCL1	0.028^*^	2.472^*^	0.254	1.570	0.723	0.675
CXCL11	0.035^*^	1.401^*^	0.217	1.227	0.795	0.651
CCL3	0.031^*^	2.010^*^	0.156	1.595	0.777	0.722
IL-8	0.096	1.742	0.030^*^	1.991^*^	0.759	0.905
CXCL9	0.211	12.501	0.048^*^	39.585^*^	0.750	0.722
TIMP-1	0.696	0.959	< 0.001^*^	0.718^*^	0.661	0.937

DM, dermatomyositis; ASS, anti-synthetase syndrome; HCs, healthy controls; CCL, C-C motif chemokine ligand; TNFR2, tumor necrosis factor receptor 2; CXCL, C-X-C motif chemokine ligand; IL, interleukin; IL-1ra, IL-1 receptor type 1; TIMP-1, tissue inhibitor of metalloproteinases-1.

* Adjusted P-value < 0.05 and foldchange > 1.2 or < 0.83.

Serum CCL2, CCL4, CXCL13, and TNFR2 were increased in patients with both DM and ASS. In addition, serum IL-1ra, IL-1b, CCL1, CXCL11, and CCL3 were increased in patients with DM only. Serum IL-8 and CXCL9 were increased in patients with ASS only, whereas serum TIMP-1 was decreased.

Compared with patients with ASS, a significantly increased expression of IL-1ra and TIMP-1 was observed in patients with DM (Supplementary Figures 1E,L).

### Correlation analysis

**Figure 3:**
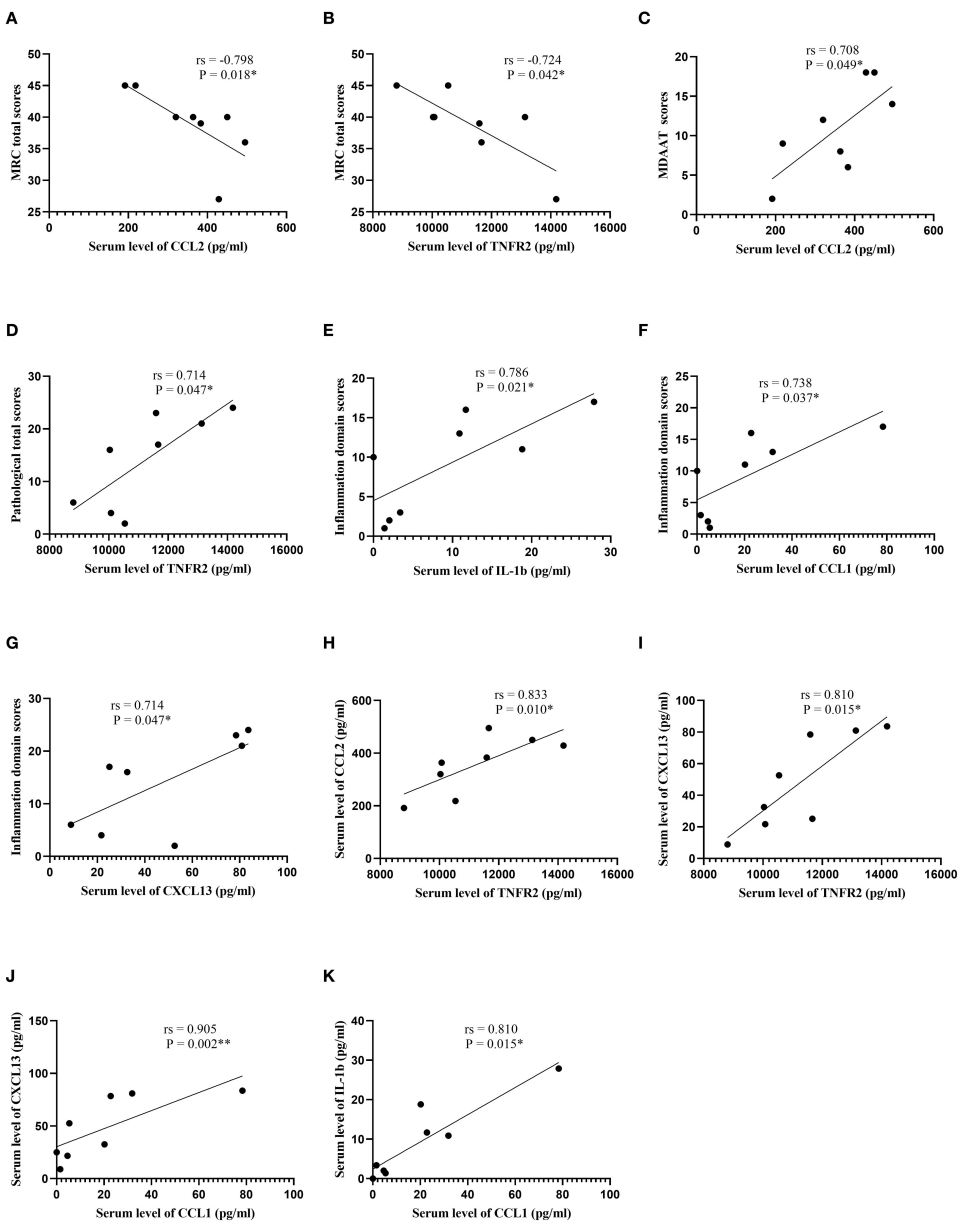
Correlation analysis in patients with dermatomyositis. **(A)** Correlation analysis between serum CCL2 levels and MRC total scores. **(B)** Correlation analysis between serum TNFR2 levels and MRC total scores. **(C)** Correlation analysis between serum CCL2 levels and MDAAT scores. **(D)** Correlation analysis between serum TNFR2 levels and pathological total scores. **(E)** Correlation analysis between serum IL-1b levels and inflammation domain scores. **(F)** Correlation analysis between serum CCL1 levels and inflammation domain scores. **(G)** Correlation analysis between serum CXCL13 levels and inflammation domain scores. **(H)** Correlation analysis between serum TNFR2 levels and serum CCL2 levels. **(I)** Correlation analysis between serum TNFR2 levels and serum CXCL13 levels. **(J)** Correlation analysis between serum CCL1 levels and serum CXCL13 levels. **(K)** Correlation analysis between serum CCL1 levels and serum IL-1b levels. CCL, C-C motif chemokine ligand; MRC, Medical Research Council; TNFR2, tumor necrosis factor receptor 2; MDAAT, Myositis Disease Activity Assessment Tool; IL, interleukin; CXCL, C-X-C motif chemokine ligand. ^*^P < 0.05; ^**^P < 0.01.

In patients with DM, serum CCL2 and TNFR2 were inversely correlated with MRC total scores (*r*s = −0.798, *P* = 0.018; *r*s = −0.724, *P* = 0.042, respectively; Supplementary Table 2; Figure 3). A direct correlation between serum CCL2 and MDAAT scores was detected (*r*s = 0.708, *P* = 0.049). In addition, serum TNFR2 was positively correlated with pathological total scores (*r*s = 0.714, *P* = 0.047). Serum IL-1b, CCL1, and CXCL13 were positively correlated with inflammation domain score (*r*s = 0.786, *P* = 0.021; *r*s = 0.738, *P* = 0.037; *r*s = 0.714, *P* = 0.047, respectively). Serum TNFR2 was positively correlated with CCL2 and CXCL13 (*r*s = 0.833, *P* = 0.010; *r*s = 0.810, *P* = 0.015, respectively). Serum CCL1 was positively correlated with IL-1b and CXCL13 (*r*s = 0.810, *P* = 0.015; *r*s = 0.905, *P* = 0.002, respectively).

**Figure 4:**
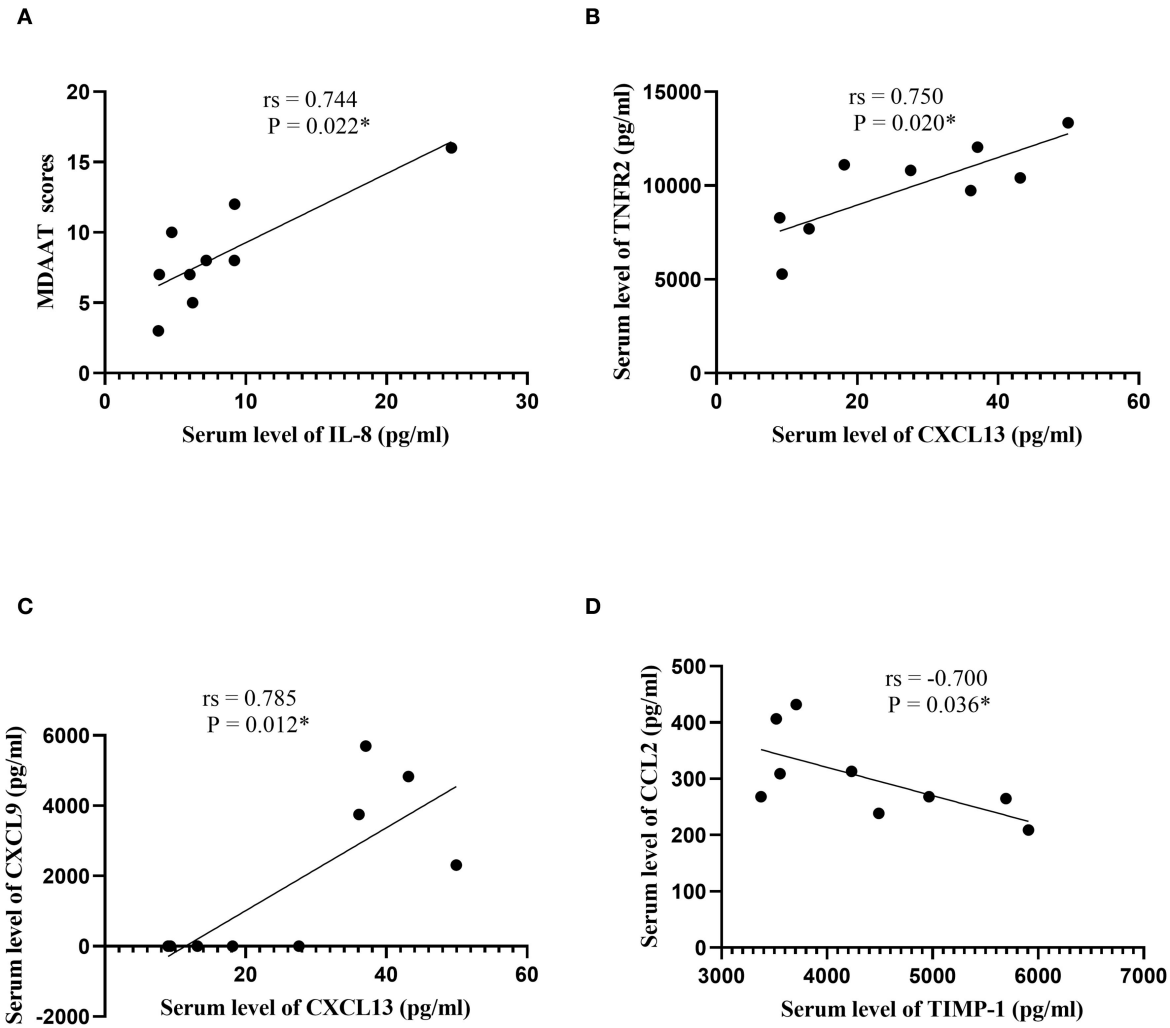
Correlation analysis in patients with the anti-synthetase syndrome. **(A)** Correlation analysis between serum IL-8 levels and MDAAT scores. **(B)** Correlation analysis between serum CXCL13 levels and serum TNFR2 levels. **(C)** Correlation analysis between serum CXCL13 levels and serum CXCL9 levels. **(D)** Correlation analysis between serum TIMP-1 levels and serum CCL2 levels. IL, interleukin; MDAAT, Myositis Disease Activity Assessment Tool; CXCL, C-X-C motif chemokine ligand; TIMP-1, tissue inhibitor of metalloproteinases-1; CCL, C-C motif chemokine ligand. ^*^P < 0.05.

In patients with ASS, a direct correlation between serum IL-8 and MDAAT scores was observed (*r*s = 0.744, *P* = 0.022; Supplementary Table 3; Figure 4). In addition, serum CXCL13 was positively correlated with TNFR2 and CXCL9 (*r*s = 0.750, *P* = 0.020; *r*s = 0.785, *P* = 0.012, respectively). Serum TIMP-1 was negatively correlated with CCL2 (*r*s = −0.700, *P* = 0.036).

### Diagnostic performance evaluation

In ROC analysis, we determined the diagnostic accuracy of individual markers to distinguish newly diagnosed patients with DM and ASS from HCs. Serum CCL2 provided excellent discrimination of patients with DM and ASS from HCs, as indicated by AUCs > 0.90 (Table 3). Serum TNFR2 and CXCL13 also accurately distinguished patients with DM and ASS from HCs, with AUCs > 0.80. Serum IL-1ra, which was not modulated in ASS, could discriminate between patients with DM and HCs, as shown by an AUC > 0.80. Serum IL-8, which was not modulated in DM, could discriminate between patients with ASS and HCs, as shown by an AUC > 0.80. None of the other markers provided an AUC > 0.80. The ROC curves of each cytokine/chemokine are shown in Supplementary Figure 2.

**Figure 5:**
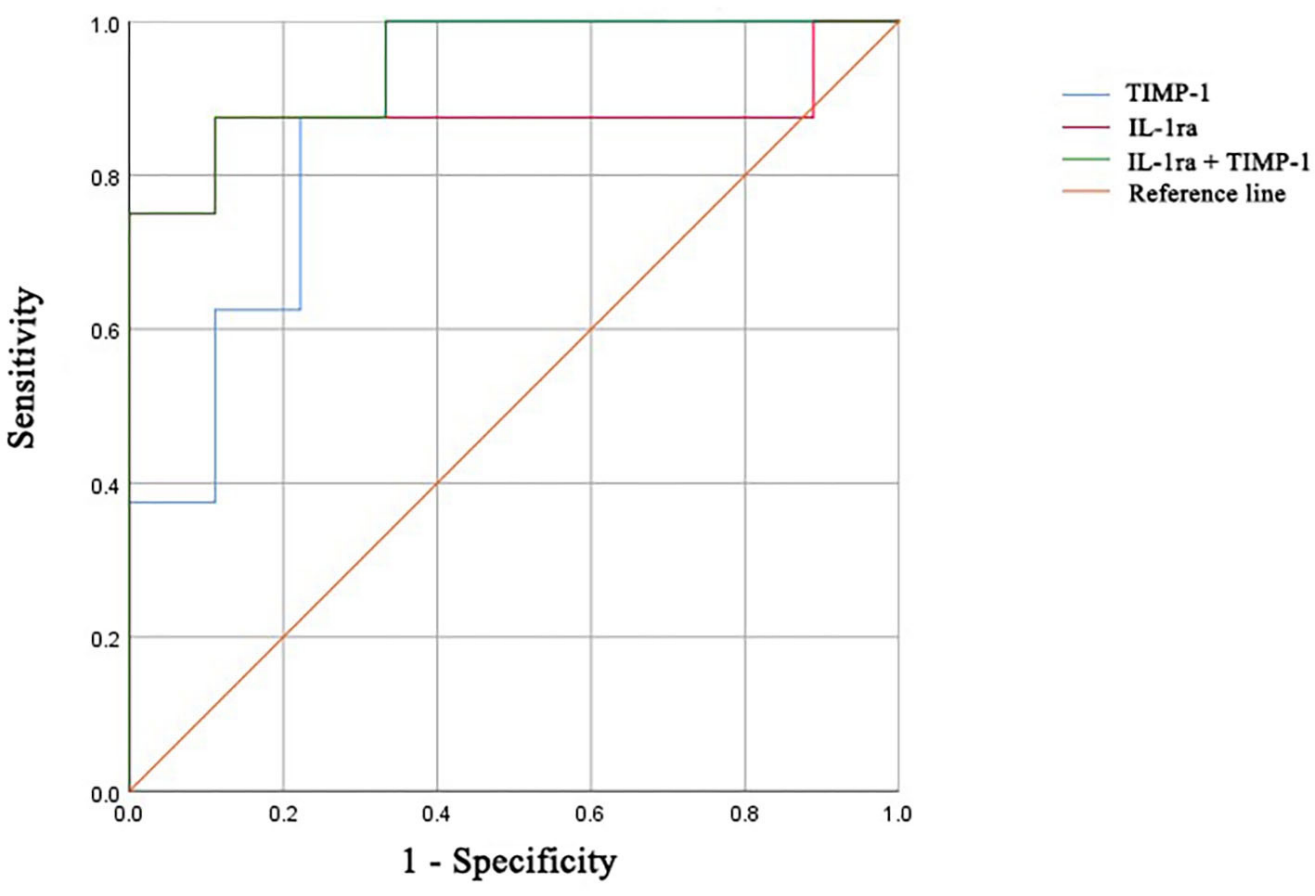
ROC curves of IL-1ra, TIMP-1, and the combination for distinguishing patients with DM from patients with ASS. ROC, receiver operating characteristic; IL-1ra, IL-1 receptor type 1; TIMP-1, tissue inhibitor of metalloproteinases-1; DM, dermatomyositis; ASS, anti-synthetase syndrome.

We next determined the diagnostic accuracy of individual markers in distinguishing newly diagnosed patients with DM from ASS. The AUC for IL-1ra was 0.875 (95% confidence interval, 0.667–1.000), with a sensitivity of 87.5% and specificity of 88.9% (Table 4 and Figure 5). The AUC for TIMP-1 was 0.875 (95% confidence interval, 0.706–1.000), with a sensitivity of 100.0% and specificity of 66.7%. The cutoff values of IL-1ra and TIMP-1 were calculated as 39.23 and 4,708 (pg/ml). Furthermore, the AUC of the combination of IL-1ra and TIMP-1 was 0.944 (95% confidence interval, 0.840–1.000), with a sensitivity of 87.5% and specificity of 88.9%.

**Table 4:** Diagnostic value of serum cytokines/chemokines for distinguishing patients with DM and ASS.

	**AUC (95%CI)**	**Youden index**	**Cut-off value**	**Sensitivity**	**Specificity**	**PPV**	**NPV**
IL-1ra	0.875 (0.667, 1.000)	0.764	39.23	87.5%	88.9%	87.5%	88.9%
TIMP-1	0.875 (0.706, 1.000)	0.667	4708	100.0%	66.7%	72.7%	100.0%
Combination	0.944 (0.840, 1.000)	0.764	–	87.5%	88.9%	87.5%	88.9%

DM, dermatomyositis; ASS, anti-synthetase syndrome; IL-1ra, IL-1 receptor type 1; TIMP-1, tissue inhibitor of metalloproteinases-1; AUC, area under the curve; CI, confidence interval; PPV, positive predictive value; NPV, negative predictive value.

## Discussion

DM and ASS are rare diseases, with incidences of 3.2–9.63 per one million persons for DM, and prevalences of 1.2–21.42 per 100,000 persons (21–23). The prevalence of ASS was estimated as 1–9 per 100,000 persons; however, no precise data on the disease incidence are available (24). It is important to distinguish these two diseases because they have different prognoses and responses to therapy. The previous study suggested that DM associated with ILD can frequently progress rapidly and have a poor outcome, whereas ASS associated with ILD usually responds to therapy (11). A few studies have reported a positive response to Janus kinase inhibitors, targeted for JAK-mediated signaling of interferon, for the treatment of refractory DM (25, 26). Considering they are distinct entities, the identification of serum biomarkers could help to better understand the pathogenesis of diseases and distinguish between DM and ASS.

Newly diagnosed and treatment-naïve patients with DM and ASS were enrolled in the present study. We also investigated and compared serum cytokine/chemokine profiles in patients with DM and ASS. Four serum markers (i.e., CCL2, CCL4, CXCL13, and TNFR2) were increased in patients with both DM and ASS. These markers modulated in both diseases further confirm that most clinical manifestations and myopathological features are indistinguishable between the two diseases. Serum CCL2, TNFR2, and CXCL13 were the most accurate markers for discriminating newly diagnosed patients with DM and ASS from HCs.

Serum CCL2 was increased in patients with DM and ASS and might provide excellent discrimination between newly diagnosed patients and HCs. CCL2 is known as a chemoattractant for mononuclear cells and T lymphocytes (27) and is selectively expressed on perimysial and perifascicular blood vessels in DM (28). High serum levels of CCL2 have been reported in DM with severe symptoms (15, 29) and with interstitial pneumonia (30). Notarnicola et al. (31) observed increased serum levels of CCL2 in patients with idiopathic inflammatory myopathies, including ASS with anti-Jo-1 antibody. It has been proposed that serum CCL2 might be a good marker for disease activity in DM (16, 29, 32–35). Serum CCL2 was closely correlated with TNFR2 in DM and TIMP-1 in ASS. In addition, serum CCL2 was strongly correlated with MRC total scores and MDAAT scores in DM. Our findings indicated that serum CCL2 was a promising biomarker for DM and ASS.

Vasculopathy is an important hallmark of systemic chronic inflammatory connective tissue diseases. As in other inflammatory conditions (33), serum CCL4 levels were increased in patients with DM and ASS. Increased serum levels of CCL4 were observed in patients with idiopathic inflammatory myopathies (31). Chronic hypoxia in macrophages increased the expression of CCL4 (36). Endothelial CCL4 expression was observed in all capillaries in DM (37), and CCL4 was present only in the perimysial inflammatory foci of a subset of DM biopsies (38). Although serum CCL4 did not show diagnostic potential comparable to that of CCL2, TNFR2, and CXCL13, the differential modulation of serum CCL4 suggested an important role in the pathological processes of DM and ASS.

Serum CXCL13 also provided excellent discrimination between newly diagnosed patients with DM and ASS from HCs. CXCL13, a central homing factor for B cells, is expressed in follicular structures and lymphocytic aggregates in juvenile DM (39); and CXCL12^+^/CXCL13^+^CD20^+^ B cells are common in ASS as well (40). It has been reported that patients with DM with severe muscle disease and global disease activity exhibited high serum levels of CXCL13 (15). Interestingly, we observed that serum CXCL13 correlated to TNFR2, CCL1, and inflammation domain scores in DM. Although elevated serum levels of CXCL13 had been observed in patients with other inflammatory diseases (41, 42), our findings indicated that serum CXCL13 was a valuable marker for diagnosing DM and ASS.

Serum TNFR2 also distinguished newly diagnosed patients with DM and ASS from HCs. TNFR2 has regulatory functions in TNF-mediated diseases and was increased in perifascicular and perimysial endothelia in patients with DM (43). TNFR2 has been related to enhanced interferon-driven inflammation in juvenile DM, especially in patients with anti-NXP2 antibodies (15). It has been proposed that serum TNFR2 was a marker for disease activity in juvenile DM (15, 44, 45). We found a significant inverse correlation between serum TNFR2 and MRC total scores, and a direct correlation between serum TNFR2 and pathological total scores in patients with DM.

Although some clinical manifestations and myopathological features were observed in both conditions, DM and ASS are two diseases with different pathogeneses and prognoses (11). Tests for anti-aminoacyl-tRNA synthetase other than anti-Jo-1 might be not accessible to all hospitals and commercial tests might lack specificity (6). To date, molecular biomarkers for the differential diagnosis of DM and ASS are still sorely lacking. Therefore, we explored if serum levels of IL-1ra and TIMP-1 could distinguish patients with DM from patients with ASS.

IL-1ra is a cytokine that controls inflammatory response in the early stage of immune activation (46), expressed in muscle fibers, inflammatory cells, and endothelial cells in DM (47). Serum IL-1ra was significantly elevated in the DM-active subset than in the DM-stable subset (48). Zhou et al. (14) also reported higher serum levels of IL-1ra in anti-MDA5 DM when compared with ASS. It was proposed that elevated serum IL-1ra in anti-NXP2 DM might switch from a controlled and protective immune role to one in which this protein promotes inflammatory tissue damage in severe cases (49). Future research on the role of IL-1ra in anti-NXP2 DM may offer new insights for the detection and treatment of patients with DM.

We also observed increased serum levels of TIMP-1 in patients with DM compared with patients with ASS. Although we were not aware of prior studies on serum TIMP-1 in DM and ASS, Nakatsuka et al. (50) reported higher serum levels of matrix metalloproteinases-7 in patients with ASS with ILD when compared with patients with any other subtypes of polymyositis/DM-ILD. Matrix metalloproteinase activity is predominantly controlled by TIMP, which is essential in the synthesis/degradation balance of the extracellular matrix. Kieseier et al. (51) found unchanged expression of TIMP-1 in muscle biopsies from patients with DM/polymyositis. Further research on the changes of TIMP-1 in DM and ASS is needed.

There were some limitations in the present study. The first limitation of our study was the small number of patients enrolled. DM and ASS are rare diseases, which hampers the collection of large sample numbers for study purposes. In addition, because our hospital is a tertiary hospital, most of the patients visiting our center were not treatment-naïve. As a result, available samples were limited and subtle changes in serum cytokines/chemokines may have gone unnoticed. The second limitation of our study was the detection of MSAs and myositis-associated autoantibodies. Immunoprecipitation is regarded as the gold standard assay and is not available in our center. However, open muscle biopsies were performed in all patients for correct diagnosis. Third, the DM group was composed of patients with DM with anti-NXP2 antibodies and the ASS group was mainly composed of patients with ASS with anti-Jo-1 and anti-EJ antibodies. Future studies should include patients with other MSAs. Finally, the diagnostic criteria of ASS described by Selva-O'Callaghan et al. are limited. The absence of anti-aminoacyl-tRNA synthetases antibody does not preclude the presence of ASS. The 2017 ACR/EULAR (European League Against Rheumatism) criteria did not distinguish DM and ASS (52), for which reason it was not chosen in the present study. Efforts to generate new ASS diagnoses and classification criteria are needed, which may improve our understanding of ASS.

## Conclusion

Our study demonstrated that serum levels of cytokines/chemokines showed a different pattern in newly diagnosed patients with DM and ASS, and the combination of serum IL-1ra and TIMP-1 can distinguish between the two diseases.

## Data availability statement

The original contributions presented in the study are included in the article/Supplementary material, further inquiries can be directed to the corresponding author.

## Ethics statement

The studies involving human participants were reviewed and approved by the Ethics Committee at Peking University First Hospital. Written informed consent to participate in this study was provided by the participants/participants' legal guardian.

## Author contributions

YW: acquisition of data, completion of statistical analysis, drafting of the initial manuscript, and writing of the final manuscript. YiZ and YaZ: acquisition of data, study concept and design, completion of statistical analysis, and critical revision of the manuscript. YL, WenZ, MY, ZX, HH, FG, and ZW: study concept and design and critical revision of the manuscript. WeiZ and YY: data review, interpretation of results, and revision of the initial draft. All authors contributed to the article and approved the submitted version.

## Supplementary material

Supplementary Table 1 Modified muscle biopsy scoring system for DM and ASS.

Supplementary Table 2 Correlation analysis in dermatomyositis.

Supplementary Table 3 Correlation analysis in anti-synthetase syndrome.

Supplementary Figure 1 Comparison of cytokines/chemokines among DM patients, ASS patients and HCs. **(A)** Statistical analysis of serum CCL2 levels. **(B)** Statistical analysis of serum CCL4 levels. **(C)** Statistical analysis of serum TNFR2 levels. **(D)** Statistical analysis of serum CXCL13 levels. **(E)** Statistical analysis of serum IL-1ra levels. **(F)** Statistical analysis of serum IL-1b levels. **(G)** Statistical analysis of serum CCL1 levels. **(H)** Statistical analysis of serum CXCL11 levels. **(I)** Statistical analysis of serum CCL3 levels. **(J)** Statistical analysis of serum IL-8 levels. **(K)** Statistical analysis of serum CXCL9 levels. **(L)** Statistical analysis of serum TIMP-1 levels. DM, dermatomyositis; ASS, anti-synthetase syndrome; HCs, healthy controls; CCL, C-C motif chemokine ligand; TNFR2, tumor necrosis factor receptor 2; CXCL, C-X-C motif chemokine ligand; IL, interleukin; IL-1ra, IL-1 receptor type 1; TIMP-1, tissue inhibitor of metalloproteinases-1. ^*^*P* < 0.05; ^**^*P* < 0.01; ^***^*P* < 0.001.

Supplementary Figure 2 Receiver operating characteristic curves of each cytokine/chemokine. **(A)** Receiver operating characteristic curves of serum CCL2. **(B)** Receiver operating characteristic curves of serum CCL4. **(C)** Receiver operating characteristic curves of serum TNFR2. **(D)** Receiver operating characteristic curves of serum CXCL13. **(E)** Receiver operating characteristic curves of serum IL-1ra. **(F)** Receiver operating characteristic curves of serum IL-1b. **(G)** Receiver operating characteristic curves of serum CCL1. **(H)** Receiver operating characteristic curves of serum CXCL11. **(I)** Receiver operating characteristic curves of serum CCL3. **(J)** Receiver operating characteristic curves of serum IL-8. **(K)** Receiver operating characteristic curves of serum CXCL9. **(L)** Receiver operating characteristic curves of serum TIMP-1. CCL, C-C motif chemokine ligand; TNFR2, tumor necrosis factor receptor 2; CXCL, C-X-C motif chemokine ligand; IL, interleukin; IL-1ra, IL-1 receptor type 1; TIMP-1, tissue inhibitor of metalloproteinases-1.
