# Supplementary material for: Comparison of cytokine/chemokine profiles between dermatomyositis and anti-synthetase syndrome
Source: Front Neurol. 2022 Dec 8;13:1042580. doi: 10.3389/fneur.2022.1042580 (PMC9772994; doi:10.3389/fneur.2022.1042580)
Supplement: Supplementary Table 1 — Modified muscle biopsy scoring system for DM and ASS. [file Table_1.DOCX]

Supplementary table 1. Modified muscle biopsy scoring system for DM and ASS.

| Pathology domain | Score/evaluation criteria |
| --- | --- |
| Muscle Fiber Domain | Maximum scores = 8 |
| Necrotic fiber | 0 = absence, 1 = isolated/sporadic 1-3 per 20×, 2 = scattered ≥ 4 or more in adjacent fiber, or clustered, or > 3 per 20× |
| Regenerating fiber | 0 ≤ 6 per 20×, 1 ≥ 6 per 20× |
| Atrophic fiber away from  perifascicular area | 0 = absence, 1 = isolated/sporadic 1-3 per 20×, 2 = scattered ≥ 4 or more in adjacent fiber, or clustered, or > 3 per 20× |
| Perifascicular atrophy | 0 = absence, 1 = affecting 1-2 fascicle (s), 2 = affecting > 2 fascicles |
| Fiber with internalized nuclei >  3% | 0 ≤ 3%, 1 > 3% |
| Inflammation domain | Maximum scores = 18 |
| Endomysial, perimysial and  perivascular CD3^+^ cell infiltration | 0 = none or < 4 cells in 20× field, 1 = 4-20 cells in a 20× field or a cluster of ≥ 10 cells, 2 = ≥ 2 clusters in whole biopsy and/or diffusely infiltrating ie. > 20 cells in a 20× field (separate scores for endomysial, perimysial and perivascular infiltration) |
| Endomysial, perimysial and  perivascular CD20^+^ cell infiltration |  |
| Endomysial, perimysial and  perivascular CD68^+^ cell infiltration |  |
| Connective tissue domain | Maximum scores = 3 |
| Any endomysial fibrosis | 0 = absence, 1 = presence |
| Any perimysial fibrosis |  |
| Perimysial connective tissue  fragmentation |  |
| Vascular domain | Maximum scores = 5 |
| Arterial abnormality | Mural thickening and/or endothelial swelling and/or transmural inflammation in arteries/arterioles. 0 = absence, 1 = presence |
| Infarction | Well demarcated regional loss of muscle fiber nuclei and loss of normal cytoarchitecture.0 = absence, 1 = presence |
| MAC^+^ capillaries | 0 = none in 20× field; 1= ≤ 5 in a 20× field; 2 = 6-10 in a 20× field; 3 = >10 in a 20× field |

DM, dermatomyositis; ASS, anti-synthetase syndrome; MAC, membrane attack complex.
