# Supplementary material for: Comparison of cytokine/chemokine profiles between dermatomyositis and anti-synthetase syndrome
Source: Front Neurol. 2022 Dec 8;13:1042580. doi: 10.3389/fneur.2022.1042580 (PMC9772994; doi:10.3389/fneur.2022.1042580)
Supplement: Supplementary Table 2 — Correlation analysis in dermatomyositis. [file Table_2.docx]

Supplementary Table 2. Correlation analysis in dermatomyositis

|  |  | TNFR2 | CXCL11 | CCL2 | IL-1b | CCL1 | CXCL13 | IL-1ra | CCL3 | CCL4 |
| --- | --- | --- | --- | --- | --- | --- | --- | --- | --- | --- |
| Clinical indicators |  |  |  |  |  |  |  |  |  |  |
| Peak CK | rs | 0.000 | -0.619 | 0.405 | 0.048 | -0.095 | -0.190 | 0.462 | -0.048 | -0.690 |
|  | P | 1.000 | 0.102 | 0.320 | 0.911 | 0.823 | 0.651 | 0.078 | 0.911 | 0.058 |
| MRC total scores | rs | -0.724 | 0.442 | -0.798 | -0.344 | -0.344 | -0.491 | -0.503 | 0.356 | 0.356 |
|  | P | 0.042* | 0.273 | 0.018* | 0.404 | 0.404 | 0.217 | 0.204 | 0.387 | 0.387 |
| MDAAT scores | rs | 0.683 | 0.317 | 0.708 | 0.024 | 0.171 | 0.439 | 0.293 | 0.043 | 0.437 |
|  | P | 0.062 | 0.444 | 0.049* | 0.954 | 0.686 | 0.276 | 0.482 | 0.142 | 0.089 |
| Pathological scores |  |  |  |  |  |  |  |  |  |  |
| Muscle fiber Domain | rs | 0.687 | 0.061 | 0.651 | 0.233 | 0.282 | 0.503 | -0.110 | -0.393 | 0.319 |
|  | P | 0.060 | 0.885 | 0.081 | 0.578 | 0.498 | 0.204 | 0.795 | 0.336 | 0.441 |
| Connective tissue domain | rs | 0.358 | 0.140 | 0.536 | 0.294 | 0.294 | 0.345 | 0.370 | -0.600 | -0.268 |
|  | P | 0.385 | 0.740 | 0.171 | 0.480 | 0.480 | 0.403 | 0.366 | 0.116 | 0.521 |
| Vascular domain | rs | 0.426 | -0.601 | 0.451 | 0.501 | 0.551 | 0.426 | 0.501 | 0.225 | 0.050 |
|  | P | 0.293 | 0.115 | 0.263 | 0.206 | 0.157 | 0.293 | 0.206 | 0.592 | 0.906 |
| Inflammation domain | rs | 0.619 | -0.262 | 0.548 | 0.786 | 0.738 | 0.714 | 0.310 | -0.143 | 0.000 |
|  | P | 0.102 | 0.531 | 0.160 | 0.021* | 0.037* | 0.047* | 0.456 | 0.736 | 1.000 |
| Total scores | rs | 0.714 | -0.214 | 0.667 | 0.643 | 0.643 | 0.690 | 0.286 | -0.238 | 0.071 |
|  | P | 0.047* | 0.610 | 0.071 | 0.086 | 0.086 | 0.058 | 0.493 | 0.570 | 0.867 |
| Serum cytokines/chemokines |  |  |  |  |  |  |  |  |  |  |
| TNFR2 | rs | - | 0.143 | 0.833 | 0.190 | 0.571 | 0.810 | 0.429 | -0.690 | 0.024 |
|  | P | - | 0.736 | 0.010* | 0.651 | 0.139 | 0.015* | 0.289 | 0.058 | 0.955 |
| CXCL11 | rs | 0.143 | - | -0.095 | -0.333 | -0.048 | 0.143 | -0.500 | -0.500 | 0.571 |
|  | P | 0.736 | - | 0.823 | 0.420 | 0.911 | 0.736 | 0.207 | 0.207 | 0.139 |
| CCL2 | rs | 0.833 | -0.095 | - | 0.000 | 0.238 | 0.476 | 0.500 | -0.690 | -0.095 |
|  | P | 0.010* | 0.823 | - | 1.000 | 0.570 | 0.233 | 0.207 | 0.058 | 0.823 |
| IL-1b | rs | 0.190 | -0.333 | 0.000 | - | 0.810 | 0.571 | 0.310 | 0.262 | -0.190 |
|  | P | 0.651 | 0.420 | 1.000 | - | 0.015* | 0.139 | 0.456 | 0.531 | 0.651 |
| CCL1 | rs | 0.571 | -0.048 | 0.238 | 0.810 | - | 0.905 | 0.405 | -0.119 | -0.048 |
|  | P | 0.139 | 0.911 | 0.570 | 0.015* | - | 0.002** | 0.320 | 0.779 | 0.911 |
| CXCL13 | rs | 0.810 | 0.143 | 0.476 | 0.571 | 0.905 | - | 0.310 | -0.476 | 0.000 |
|  | P | 0.015* | 0.736 | 0.233 | 0.139 | 0.002** | - | 0.456 | 0.233 | 1.000 |
| IL-1ra | rs | 0.429 | -0.500 | 0.500 | 0.310 | 0.405 | 0.310 | - | -0.119 | -0.619 |
|  | P | 0.289 | 0.207 | 0.207 | 0.456 | 0.320 | 0.456 | - | 0.779 | 0.102 |
| CCL3 | rs | -0.690 | -0.500 | -0.690 | 0.262 | -0.119 | -0.476 | -0.119 | - | 0.048 |
|  | P | 0.058 | 0.207 | 0.058 | 0.531 | 0.779 | 0.233 | 0.779 | - | 0.911 |
| CCL4 | rs | 0.024 | 0.571 | -0.095 | -0.190 | -0.048 | 0.000 | -0.619 | 0.048 | - |
|  | P | 0.955 | 0.139 | 0.823 | 0.651 | 0.911 | 1.000 | 0.102 | 0.911 | - |

CK, creation kinase; MRC, Medical Research Council; MDAAT, myositis disease activity assessment tool; TNFR2, tumor necrosis factor receptor 2; CXCL, C-X-C motif chemokine ligand; CCL, C-C motif chemokine ligand; IL, interleukin; IL-1ra, IL-1 receptor type 1. rs, Spearman’s correlation coefficient; P, P value. *, P < 0.05; **, P < 0.01.
