# Supplementary material for: Comparison of cytokine/chemokine profiles between dermatomyositis and anti-synthetase syndrome
Source: Front Neurol. 2022 Dec 8;13:1042580. doi: 10.3389/fneur.2022.1042580 (PMC9772994; doi:10.3389/fneur.2022.1042580)
Supplement: Supplementary Table 3 — Correlation analysis in anti-synthetase syndrome. [file Table_3.pdf]

Supplementary Table 3. Correlation analysis in anti-synthetase syndrome

		CXCL9	IL-8	CXCL13	CCL4	CCL2	TIMP-1	TNFR2
Clinical indicators								
Peak CK	rs	0.365	0.350	0.150	0.383	0.533	-0.283	0.017
	P	0.334	0.356	0.700	0.308	0.139	0.460	0.966
MRC total scores	rs	-0.305	-0.017	-0.380	-0.267	-0.363	0.194	-0.110
	P	0.425	0.966	0.313	0.488	0.337	0.617	0.779
MDAAT scores	rs	0.553	0.744	0.513	0.633	0.231	0.248	0.342
	P	0.123	0.022*	0.158	0.057	0.550	0.520	0.368
Pathological scores								
Muscle fiber Domain	rs	0.193	-0.277	0.134	0.319	0.571	-0.445	-0.067
	P	0.618	0.470	0.730	0.402	0.108	0.230	0.864
Connective tissue domain	rs	0.243	0.027	0.168	0.275	0.612	-0.523	0.018
	P	0.529	0.946	0.665	0.474	0.080	0.148	0.964
Vascular domain	rs	-0.345	-0.254	-0.166	0.149	0.533	-0.411	-0.122
	P	0.363	0.510	0.669	0.703	0.139	0.272	0.754
Inflammation domain	rs	0.204	0.170	0.424	0.339	0.458	-0.085	0.356
	P	0.598	0.663	0.256	0.372	0.215	0.828	0.347
Total scores	rs	-0.037	-0.025	0.075	0.259	0.628	-0.452	-0.025
	P	0.925	0.949	0.847	0.500	0.070	0.222	0.949
Serum cytokines/chemokines								
CXCL9	rs	-	-0.201	0.785	0.383	0.146	0.237	0.475
	P	-	0.604	0.012*	0.308	0.708	0.539	0.197
IL-8	rs	-0.201	-	0.033	-0.267	-0.067	-0.067	0.167
	P	0.604	-	0.932	0.488	0.865	0.865	0.668
CXCL13	rs	0.785	0.033	-	0.633	0.000	0.550	0.750
	P	0.012*	0.932	-	0.067	1.000	0.125	0.020*
CCL4	rs	0.383	-0.267	0.633	-	-0.083	0.367	0.517
	P	0.308	0.488	0.067	-	0.831	0.332	0.154
CCL2	rs	0.146	-0.067	0.000	-0.083	-	-0.700	-0.217

	P	0.708	0.865	1.000	0.831	-	0.036*	0.576
TIMP-1	rs	0.237	-0.067	0.550	0.367	-0.700	-	0.667
	P	0.539	0.865	0.125	0.332	0.036*	-	0.050
TNFR2	rs	0.475	0.167	0.750	0.517	-0.217	0.667	-
	P	0.197	0.668	0.020*	0.154	0.576	0.059	-

CK, creation kinase; MRC, Medical Research Council; MDAAT, myositis disease activity assessment tool; CXCL, C-X-C motif chemokine ligand; IL, interleukin; CCL, C-C motif chemokine ligand; TIMP-1, tissue inhibitor of metalloproteinases-1; TNFR2, tumor necrosis factor receptor 2. rs, Spearman’s correlation coefficient; p, p value. *, $P < 0.05$.
