# Supplementary figures and images for: Comparison of cytokine/chemokine profiles between dermatomyositis and anti-synthetase syndrome
Source: Front Neurol. 2022 Dec 8;13:1042580. doi: 10.3389/fneur.2022.1042580 (PMC9772994; doi:10.3389/fneur.2022.1042580)

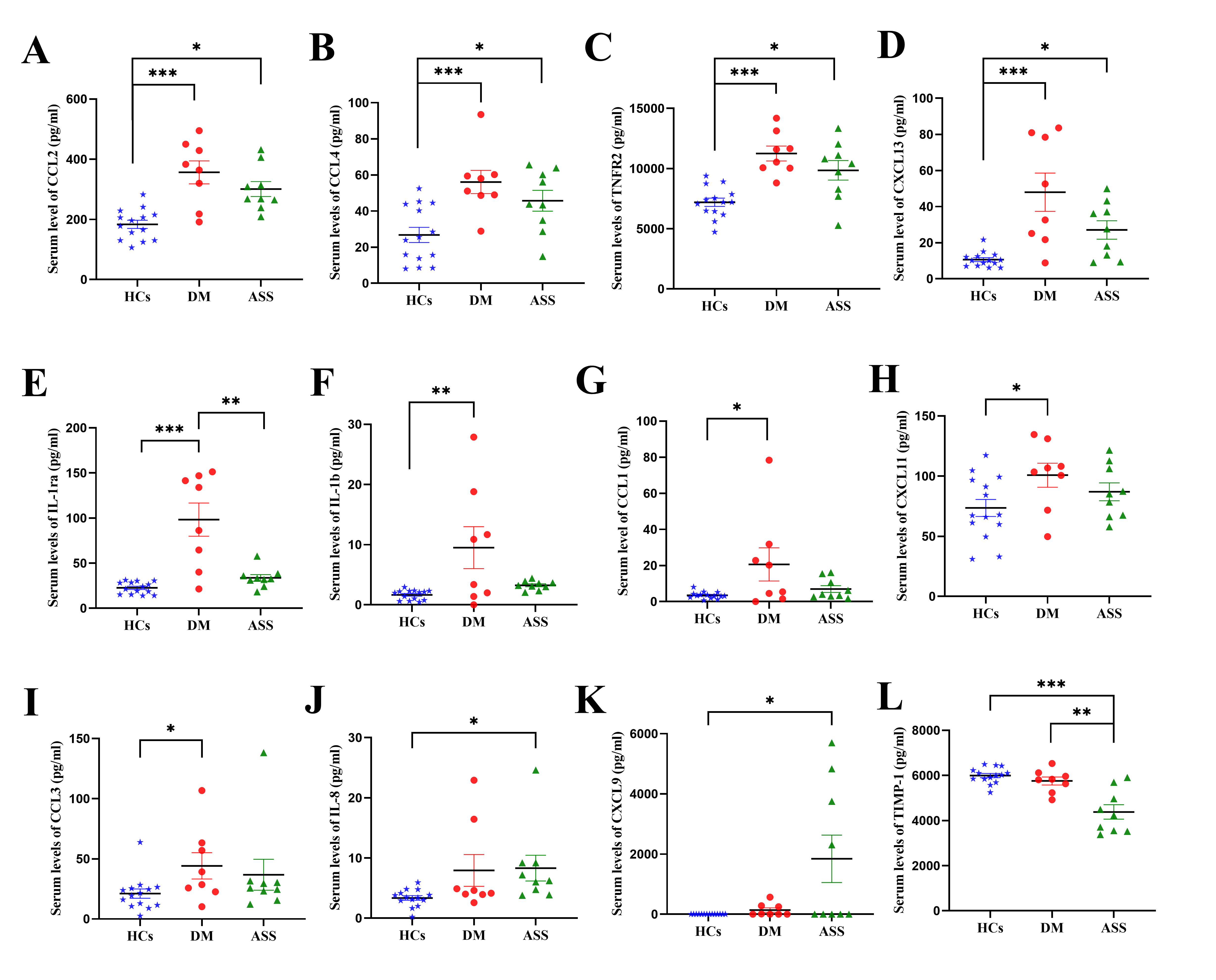

Supplement: Supplementary Figure 1 — Comparison of cytokines/chemokines among DM patients, ASS patients and HCs. (A) Statistical analysis of serum CCL2 levels. (B) Statistical analysis of serum CCL4 levels. (C) Statistical analysis of serum TNFR2 levels. (D) Statistical analysis of serum CXCL13 levels. (E) Statistical analysis of serum IL-1ra levels. (F) Statistical analysis of serum IL-1b levels. (G) Statistical analysis of serum CCL1 levels. (H) Statistical analysis of serum CXCL11 levels. (I) Statistical analysis of serum CCL3 levels. (J) Statistical analysis of serum IL-8 levels. (K) Statistical analysis of serum CXCL9 levels. (L) Statistical analysis of serum TIMP-1 levels. DM, dermatomyositis; ASS, anti-synthetase syndrome; HCs, healthy controls; CCL, C-C motif chemokine ligand; TNFR2, tumor necrosis factor receptor 2; CXCL, C-X-C motif chemokine ligand; IL, interleukin; IL-1ra, IL-1 receptor type 1; TIMP-1, tissue inhibitor of metalloproteinases-1. *P < 0.05; **P < 0.01; ***P < 0.001. [file Image_1.TIF]

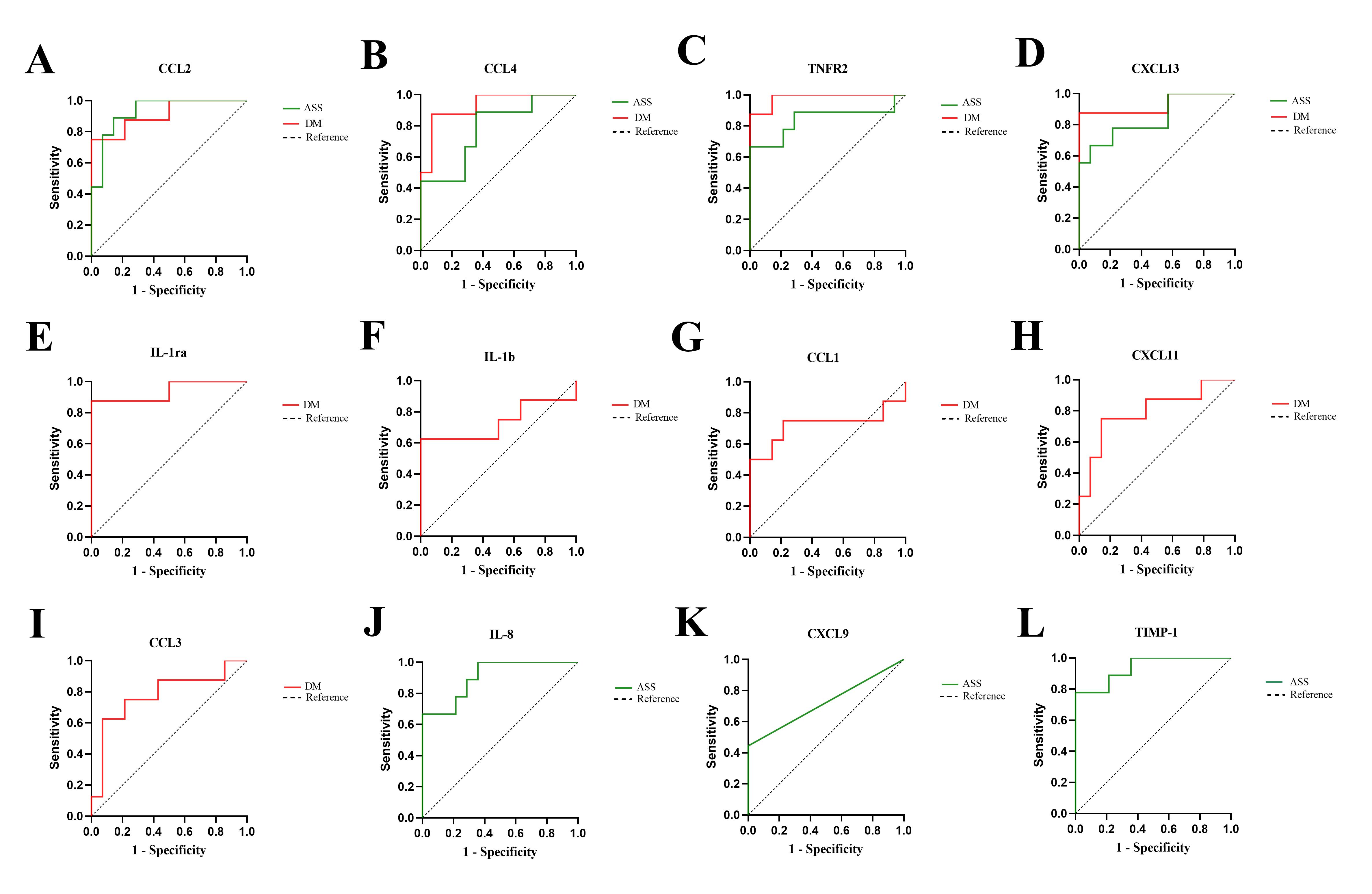

Supplement: Supplementary Figure 2 — Receiver operating characteristic curves of each cytokine/chemokine. (A) Receiver operating characteristic curves of serum CCL2. (B) Receiver operating characteristic curves of serum CCL4. (C) Receiver operating characteristic curves of serum TNFR2. (D) Receiver operating characteristic curves of serum CXCL13. (E) Receiver operating characteristic curves of serum IL-1ra. (F) Receiver operating characteristic curves of serum IL-1b. (G) Receiver operating characteristic curves of serum CCL1. (H) Receiver operating characteristic curves of serum CXCL11. (I) Receiver operating characteristic curves of serum CCL3. (J) Receiver operating characteristic curves of serum IL-8. (K) Receiver operating characteristic curves of serum CXCL9. (L) Receiver operating characteristic curves of serum TIMP-1. CCL, C-C motif chemokine ligand; TNFR2, tumor necrosis factor receptor 2; CXCL, C-X-C motif chemokine ligand; IL, interleukin; IL-1ra, IL-1 receptor type 1; TIMP-1, tissue inhibitor of metalloproteinases-1. [file Image_2.TIF]
